# Supplementary figures and images for: Cellular sialoglycans are differentially required for endosomal and cell-surface entry of SARS-CoV-2 in lung cell lines
Source: PLoS Pathog. 2024 Dec 3;20(12):e1012365. doi: 10.1371/journal.ppat.1012365 (PMC11642992; doi:10.1371/journal.ppat.1012365)

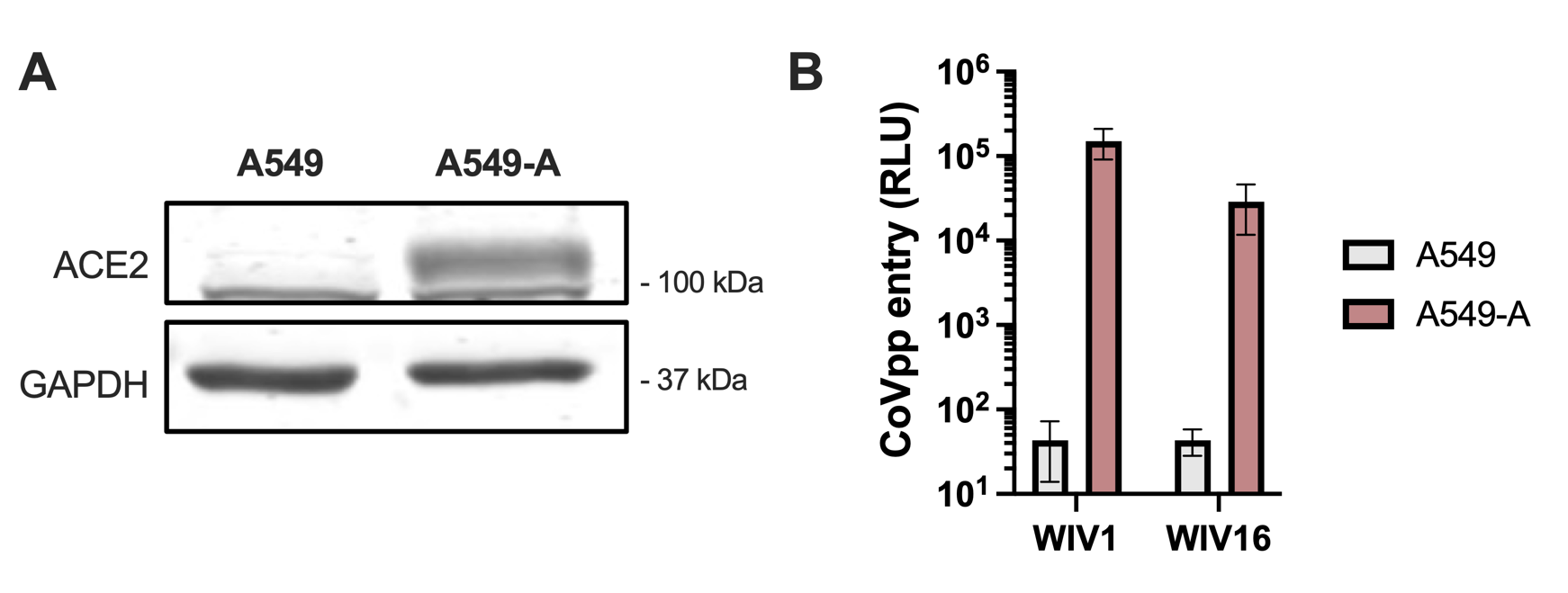

Supplement: S1 Fig — (A) Western blot assessing ACE2 expression in parental A549 cells and A549-ACE2 cells. (B) A549 or A549-ACE2 cells were inoculated with lentiviral particles pseudotyped with the spike proteins of WIV1-CoV or WIV16-CoV for 2 h, then incubated for an additional 72 h, at which point luciferase activity was measured to assess pseudoparticle entry. The data are expressed as fold change relative to the luciferase signal obtained with no envelope. Graphs show mean +/- SEM from three independent experiments performed in triplicate. (TIF) [file ppat.1012365.s001.tif]

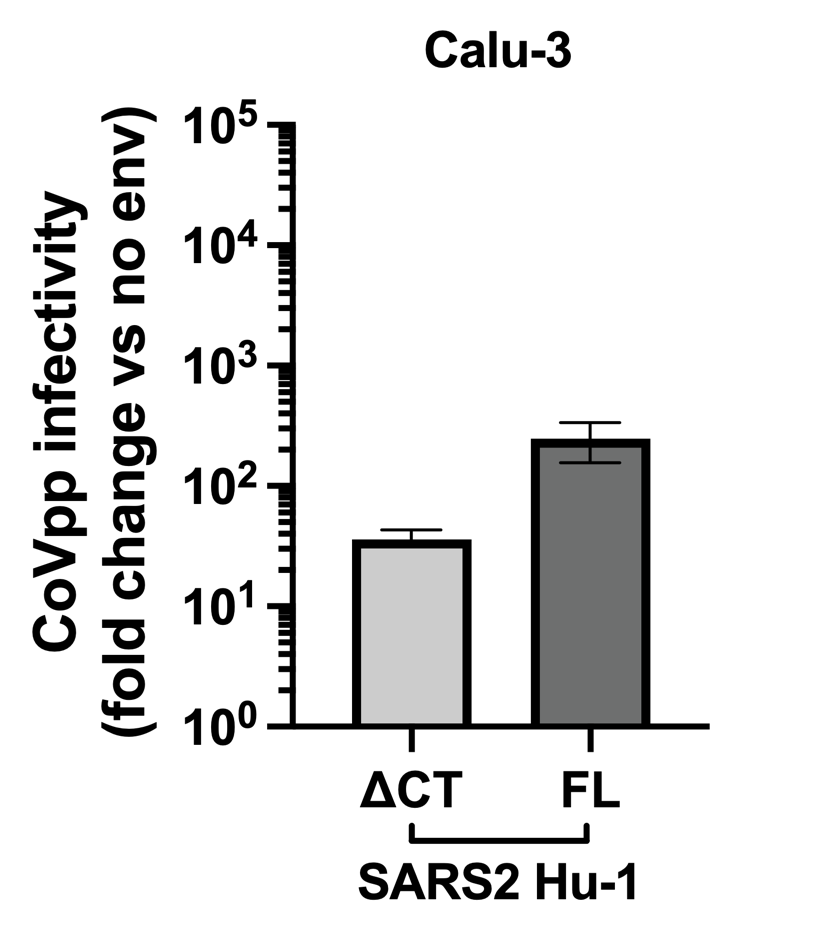

Supplement: S2 Fig — Calu-3 cells were inoculated with lentiviral particles pseudotyped with the spike proteins of SARS-CoV-2 Hu-1 (full-length or C-terminally truncated) or pseudoparticles lacking envelope protein (no env) for 2 h, then incubated for an additional 72 h, at which point luciferase activity was measured to assess pseudoparticle entry. The data are expressed as fold change relative to the luciferase signal obtained with no envelope. Graphs show mean +/- SEM from three independent experiments performed in triplicate. (TIF) [file ppat.1012365.s002.tif]

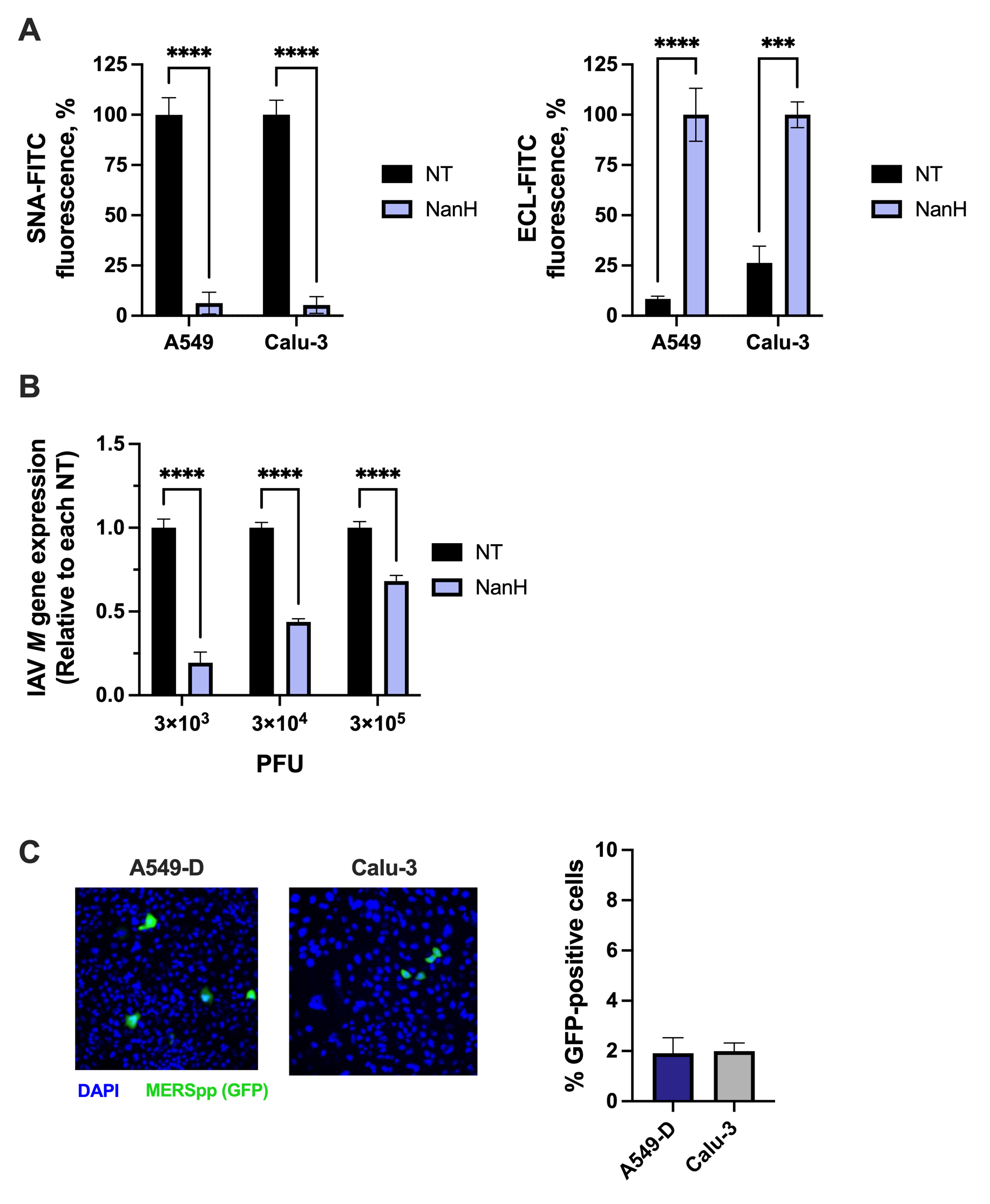

Supplement: S3 Fig — (A-B) A549 cells or Calu-3 cells were pre-treated with NanH diluted to 50 μg/mL in serum-free media for 30 minutes at 37°C. NanH-treated cells were stained with SNA-FITC (binds sialic acid) or ECL-FITC (binds galactose) diluted to final concentration of 20 μg/mL in PBS, then washed with PBS and imaged by fluorescence microscopy (representative images shown in Fig 5A). The mean FITC fluorescence intensity was calculated using ImageJ and is plotted in the graphs. (A) NanH-treated Calu-3 cells were infected with the indicated doses of IAV. At 8 hpi, cellular lysates were collected and IAV RNA (encoding the M gene) was assessed by RT-qPCR. The data were normalized to actin and are expressed relative to the non-treated condition. (C) A549-D or Calu-3 cells were inoculated with MERS-CoVpp, and GFP reporter expression was assessed 72 hours later. Representative images are shown (20X magnification). The percentage of infected cells in each condition was determined using ImageJ. Graphs show mean +/- SD (A,C) or SEM (B) from two or three independent experiments performed in triplicate. Statistical significance was assessed by two-way ANOVA (***p<0.001; ****p<0.0001). (TIF) [file ppat.1012365.s003.tif]

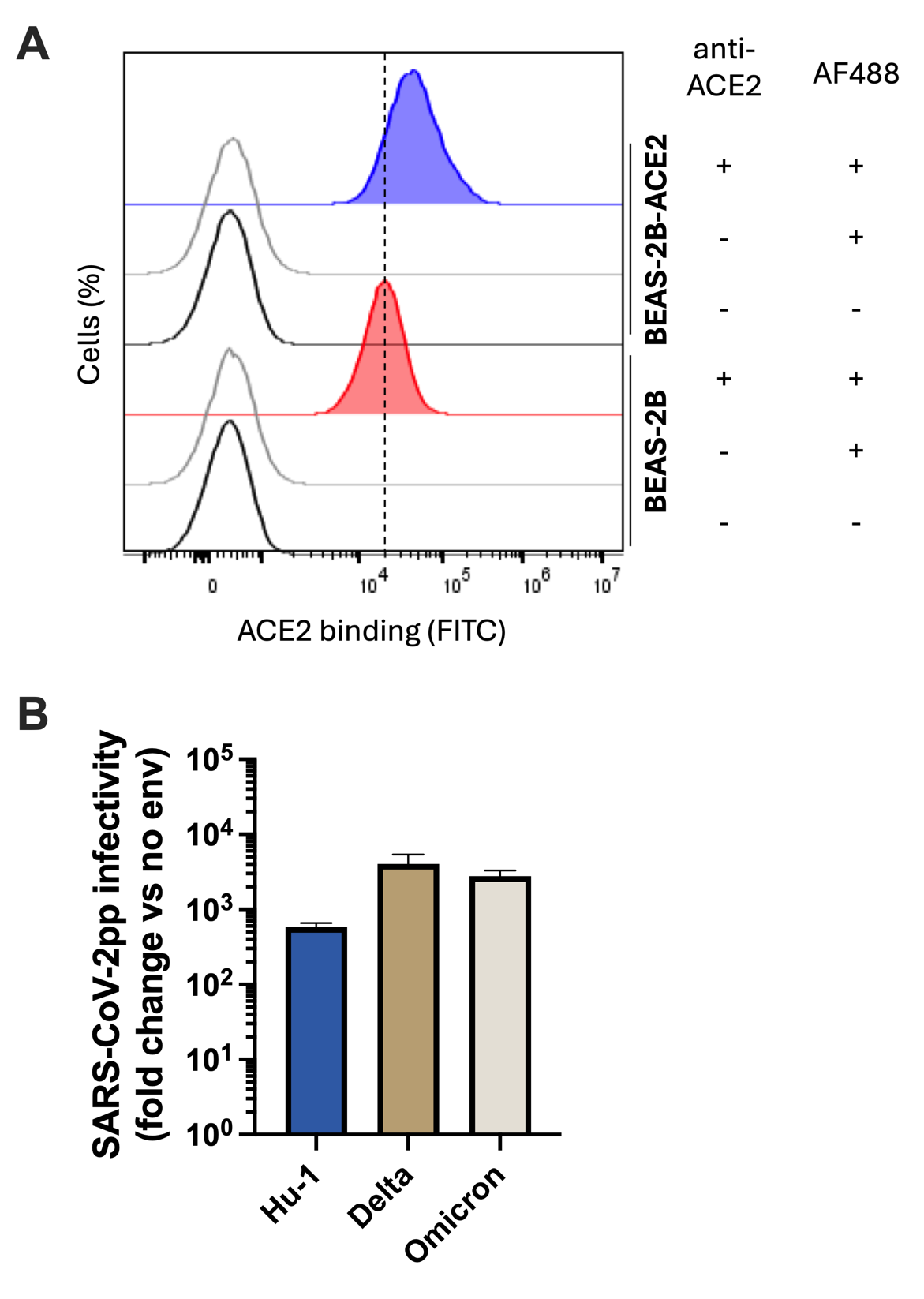

Supplement: S4 Fig — (A-B) BEAS-2B cells were transduced to stably overexpress ACE2, which was confirmed using flow cytometry (A). (B) BEAS-2B-ACE2 cells were inoculated with lentiviral particles pseudotyped with the spike proteins of SARS-CoV-2 Hu-1, SARS-CoV-2 delta or SARS-CoV-2 omicron, or pseudoparticles lacking envelope protein (no env) for 2 h, then incubated for an additional 72 h, at which point luciferase activity was measured to assess pseudoparticle entry. The data are expressed as fold change relative to the luciferase signal obtained with no envelope. Graphs show mean +/- SEM from three independent experiments performed in triplicate. (TIF) [file ppat.1012365.s004.tif]

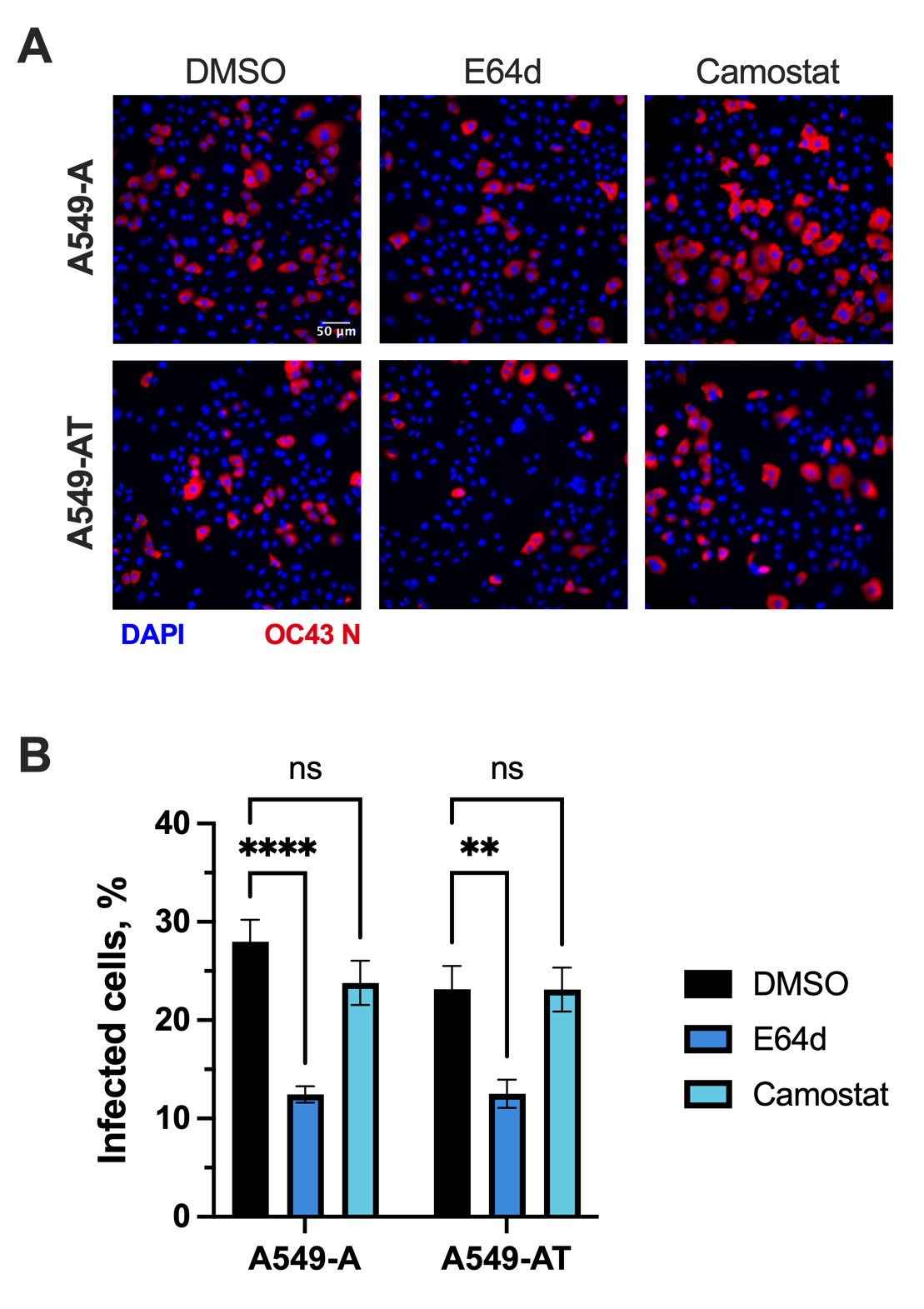

Supplement: S5 Fig — (A-B) A549-A or A549-AT cells were pre-treated for 1 h at 37°C with DMSO, camostat (25 μM) or E64d (10 μM) diluted in media, then infected with HCoV-OC43 at an MOI of 0.5 ffu/cell for 1 h at 37°C. Cells were then incubated in complete media for an additional 7 h, at which point they were fixed and processed for immunofluorescence microscopy using an antibody against the HCoV-OC43 N protein. (A) Representative images are shown (20X magnification; scale bar, 50 μm). (B) The percentage of infected cells in each condition was determined using ImageJ. Graphs show mean +/- SEM from three independent experiments performed in triplicate. Statistical significance was assessed by two-way ANOVA (ns, not significant; ***p<0.001; ****p<0.0001). (TIF) [file ppat.1012365.s005.tif]
